# Antibacterial Surgical Sutures Developed Using Electrostatic Yarn Wrapping Technology

**DOI:** 10.3390/jfb14050248

**Published:** 2023-04-28

**Authors:** Ching-Wen Lou, Chun-Yu Hung, Mengdan Wei, Tingting Li, Bing-Chiuan Shiu, Jia-Horng Lin

**Affiliations:** 1 Fujian Key Laboratory of Novel Functional Textile Fibers and Materials, Minjiang University, Fuzhou 350108, China; 2 Department of Orthopaedic Surgery, Chang Gung Memorial Hospital, Yunlin 638, Taiwan; 3 Innovation Platform of Intelligent and Energy-Saving Textiles, School of Textile Science and Engineering, Tiangong University, Tianjin 300387, China; 4 Department of Bioinformatics and Medical Engineering, Asia University, Taichung City 413305, Taiwan; 5 Department of Medical Research, China Medical University Hospital, China Medical University, Taichung City 404333, Taiwan; 6 Department of Orthopaedic Surgery, Jen-Ai Hospital, Taichung City 412, Taiwan; 7 Tianjin and Ministry of Education Key Laboratory for Advanced Textile Composite Materials, Tiangong University, Tianjin 300387, China; 8 College of Material and Chemical Engineering, Minjiang University, Fuzhou 350108, China; 9 Laboratory of Fiber Application and Manufacturing, Department of Fiber and Composite Materials, Feng Chia University, Taichung City 407102, Taiwan; 10 School of Chinese Medicine, China Medical University, Taichung City 404333, Taiwan

**Keywords:** HPC, zinc acetate, electrostatic wrapping yarns, surgical sutures

## Abstract

A significant amount of research has been conducted on applying functional materials as surgical sutures. Therefore, research on how to solve the shortcomings of surgical sutures through available materials has been given increasing attention. In this study, hydroxypropyl cellulose (HPC)/PVP/zinc acetate nanofibers were coated on absorbable collagen sutures using an electrostatic yarn winding technique. The metal disk of an electrostatic yarn spinning machine gathers nanofibers between two needles with positive and negative charges. By adjusting the positive and negative voltage, the liquid in the spinneret is stretched into fibers. The selected materials are toxicity free and have high biocompatibility. Test results indicate that the nanofiber membrane comprises evenly formed nanofibers despite the presence of zinc acetate. In addition, zinc acetate can effectively kill 99.9% of *E. coli* and *S. aureus.* Cell assay results indicate that HPC/PVP/Zn nanofiber membranes are not toxic; moreover, they improve cell adhesion, suggesting that the absorbable collagen surgical suture is profoundly wrapped in a nanofiber membrane that exerts antibacterial efficacy and reduces inflammation, thus providing a suitable environment for cell growth. The employment of electrostatic yarn wrapping technology is proven effective in providing surgical sutures with antibacterial efficacy and a more flexible range of functions.

## 1. Introduction

As the most abundant biomass in nature, cellulose is the most appealing natural material for the human being. Cellulose and its derivatives have a long history of application in medicine. Hydroxypropyl cellulose (HPC) is a cellulose derivative with FDA approval [1], a product of cellulose hydroxypropyl, and a macromolecular polysaccharide linked to glucose by β-1,4 glycosidic bonds [2]. Additionally, HPC is a cellulose ether, widely used in many technical fields, and high-performance HPC is widely used in food additives, thickeners, and emulsion stabilizers. In addition, HPC is used as a pharmaceutical formulation. However, cellulose does not dissolve in water or general organic solvents, which considerably restricts its application and development. Created through the hydroxypropylation of cellulose, HPC is a non-ionic cellulose ether and shows better water solubility than ordinary cellulose [3]. The incorporation of hydroxypropyl weakens the intramolecular and intermolecular hydrogen-bonding forces of cellulose, which benefits the water’s solubility to a great extent [4]. In the pyran ring structure of hydroxypropyl cellulose, there are three active hydroxyl groups, two secondary hydroxyl groups, and one primary hydroxyl group, located at the C2, C3, and C6 positions, respectively [5,6]. The reactive activity of the hydroxyl group at C2 and C6 is higher than that of the hydroxyl group at C3. According to the steric hindrance theory, the hydroxyl group at C6 is more reactive [7,8,9,10]. Additionally, HPC can be dissolved in organic solvents, such as CH_2_Cl_2_ and ethyl alcohol [11]. Exhibiting good biocompatibility [12], degradability [13], and hydrophilic performance as well as nontoxicity and a lack of harmful physical effects, HPC can adhere to wounds and is suitable for wound healing. In addition, HPC has good swelling properties, making it a popular material in the biological and medical fields. When used for electrospinning, pure HPC is difficult to form into membranes. As a result, pure HPC needs water-soluble, high-molecular compounds so spinning for its membrane-forming and filamentous properties can be implemented.

Polyvinyl pyrrolidone (PVP) is an amphiphilic polymer with high solubility, biocompatibility, film-forming abilities, and compatibility with various organic and inorganic compounds, which has inspired additional studies on PVP and electrostatic spinning [14,15,16]. Due to its excellent physical and chemical properties and high polymer compatibility, PVP has played a significant role in biological medicine, medical treatments, public health, [17,18] medical laboratory technologies, and pharmaceutical preparation, especially in targeted drug delivery systems [19]. PVP is a promising biomedical material [20]. PVP has hydrophilia and a structure consisting of a strongly polar lactam group, which enables PVP to be dissolved in water and organic solvents, such as ethanol.

In recent years, a great number of both chitosan hemostatic dressings [21] and electrospinning composite medical dressings have been developed [22]. When PVP and HPC are blended, the nanofiber membrane is much better formed via electrospinning. Featuring highly controlled costs, electrospinning is a technique that produces microfine polymer fibers and can, therefore, be upgraded easily from the laboratory level to commercial manufacturing [23]. It uses a polymer solution, or melt jet, that evaporates to form fibers ranging in diameter from nanometers to micrometers [24]. At present, electrospun fiber membrane polymers, traditionally used in biomedicine, are prepared with a single polymer. Considering the advantages and disadvantages of natural polymer and synthetic polymer, more and more studies have been conducted on mixing different polymers, considering the good biocompatibility of natural polymer and other excellent physical properties of synthetic polymer. Electrostatic spinning produces nanofiber membranes that possess the morphology and physical properties of a certain standard. The constituent materials can activate a cellular response, thereby accelerating wound healing and restricting the spread of bacteria surrounding the wounds. Electrospinning as an economical and efficient EHD method has been widely used in industry and laboratory. In this method, drug biomolecules, or preparations, are directly encased in the fibers, resulting in them being unaffected by environmental parameters while achieving release control [25]. With the advanced electric spinning equipment and plethora of studies on its materials, electrospun fiber films satisfy the basic requirements of wound care and have become a blessing for patients bearing skin wounds. In particular, PVP membranes exhibit high absorption capacity and air permeability and are qualified candidates for use as hemostatic materials in medical centers. Tamizi et al. [26] took the unique advantages of PVA, e.g., biocompatibility and nontoxicity, and combined PVP and sodium alga acid to form nanofiber membranes with a core-shell structure for drug delivery. Many researchers have employed efficient physical hybrid methods to produce antibacterial materials from PVP and other compounds. For example, Ignatova et al. [27] employed electrospinning to transform PVP and PEO/PVP solutions into PEO/PVP membranes.

As a material applied clinically with tremendous rates of use, absorbable collagen surgical sutures are nontoxic, non-irritating, and effectively antibacterial [28]. Collagen-absorbable sutures proceed with enzymatic degradation and eventually become polymers that are hydrolyzed [29]. The main properties of sutures include in vitro, in vivo, physiological load, binding capacity (KHC), biomechanical analysis, suture inspection, etc. [30]. Smrithi Padmakumar, John Joseph et al. used small-diameter electrostatic spinning “cores” with externally loaded electrostatic spinning “jackets” for suture applications [31]. In fact, collagen absorbable sutures are mechanically robust and demonstrate more minor tissue reactivity than standard silk sutures and absorbable sutures [32]. Suture materials are used to bring body tissues together until healing takes place [33]. Nonetheless, grafting absorbable collagen sutures onto the human body internally could trigger acute inflammation and breed bacteria around the wounds. Therefore, in this study, the electrospinning technique is used to wrap absorbable collagen sutures in a nanofiber membrane, thereby strengthening the antibacterial effect. Absolute ethanol is used as a solvent, where 0.5–1 g of HPC and 0.2 g of PVP are added, formulating the electrospinning mix. After the optimal mixture is acquired, zinc acetate is incorporated for antibacterial efficacy. The zinc-contained electrospinning mixtures undergo the electrospinning process to wrap collagen surgical sutures in the HPC/PVP/Zn nanofiber membrane. The MTT assay, antibacterial test, and degradability test results suggest that the surgery sutures are absorbable and antibacterial. To sum up, nanofiber membranes are composed of soluble and antibacterial fibers and thus exhibit biocompatibility and a uniform absorption performance that deprives the bacteria of adhesion sites, killing the microorganism while reducing inflammation risk and inflammatory responses in the tissues [34].

A membrane prepared via electrospinning has the characteristics of high porosity, a large specific surface area, and excellent mechanical properties. In addition to serving as a loading platform for drug delivery, growth factors, and other biomolecules, this property promotes cell adhesion, proliferation, and differentiation. Therefore, electrospinning has become an advanced wound-dressing technology. Antimicrobials are classified according to the main microorganisms they attack, such as viruses or bacteria. They are divided into two groups according to the compounds they contain. The first category is synthetic or chemical antimicrobials, such as antibiotic drugs and metals and metal oxide nanoparticles (NPs), such as silver and silver oxide. Herbal antimicrobials make up the second category. The antibacterial agent mainly studied here are the first type of metal ions [35]. With the development of the biomedical device industry, strips and glues have been used to replace suture in some cases. While there are other options, the increasing number of surgical procedures has increased the need for sutures. According to the global forecast, the growth of the sutures industry is slow and there is a shortage of new suture materials. Therefore, materials with high biocompatibility between HPC and PVP have been selected for this paper. Electrospinning was chosen because it is a technique that produces multifunctional fibers, spinning nanoscale fibers to carry more drugs, and because this study is focused on loading more antibacterial zinc. Anhydrous ethanol was used as a solvent, the addition of HPC was regulated at 0.5 g to 1 g, and the content of PVP was fixed at 0.2 g. After seeking the optimal ratio, zinc acetate was added to increase its antibacterial effects. After determining that the zinc solution could spin evenly formed nanofibers and then using the electrospinning technique, the HPC/PVP/Zn solution was coated using electrostatic yarn on absorbable collagen sutures. Because the absorbable and antibacterial effects of the surgical sutures have been identified, we perform cytotoxicity, antibacterial, and degradability tests. The aim was to coat collagen sutures with soluble, anti-bacterial fibers using the electrospinning technique and to produce a suture with high biocompatibility that can also kill microorganisms and bacteria around the wound, thus achieving an antibacterial effect.

## 2. Experimental

### 2.1. Materials

Hydroxypropyl cellulose (HPC, CAS:9004-64-2) was supplied by the Shanghai Aladdin Biochemical Technology Co., Ltd., (Shanghai, China). Polyvinyl pyrrolidone (PVP, CAS:9003-39-8) was provided by the Sigma Aldrich Shanghai Trading Co., Ltd., Shanghai, China. Zinc acetate (ZA, AR, CAS:557-34-6) was supplied by the Shanghai Maclin Biochemical Technology Co., Ltd., Shanghai, China. Absorbable collagen sutures were purchased from the Shandong Boda Medical Supplies Co., Ltd., Heze, China. Agar (Agar, BR, CAS:9002-18-0) was purchased from the Guangzhou Shuopu Biotechnology Co., Ltd., (Guangzhou, China). NaCl (NaCl, AR, CAS:7647-14-5) was purchased from the Xilong Science Co., Ltd., China. Yeast extract powders (BR) were purchased from the Nanjing Maojie Microbial Technology Co., Ltd., Nanjing, China. Peptone (Peptone, BR, 01-001) was purchased from the Beijing Aoboxing Biotechnology Co., Ltd., Beijing, China. 

### 2.2. Preparation of HPC/PVP/Zn Nanofiber Membranes

**Figure 1:**
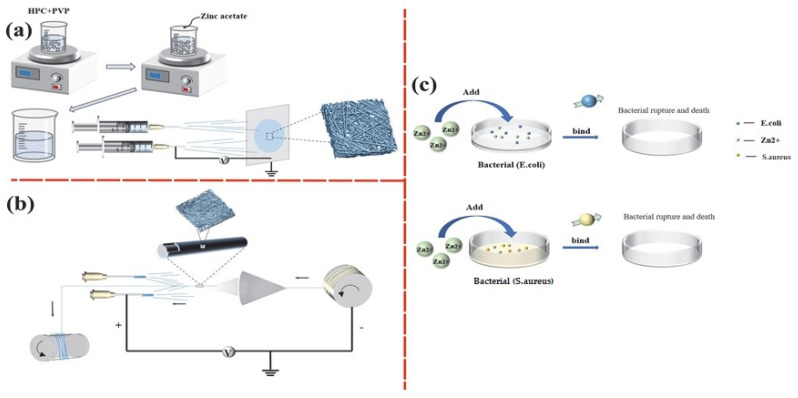
(**a**) Electrospinning process of HPC/PVP/Zn nanofibers; (**b**) the preparation of HPC/PVP/Zn-wrapped yarns; and (**c**) the antibacterial mechanisms schematic illustration of HPC/PVP/Zn nanofiber against *E. coli* or *S. aureus*.

An electrostatic spinning machine, assembled with a 10-mL injection syringe, an injection pump, a metal needle, a pressurized power supply, and a diverter, was employed (Figure 1a,b). The velocity of the injection syringe and the distance between the injection syringe and the diverter was tested. In the pilot study, 10 mL of absolute ethanol was used as the specified solvent, and PVP was selected with 2% *w*/*v* with HPC content changing to 5, 6, 7, 8, and 10% *w*/*v,* as in Table 1. The blends were infused in a beaker and magnet-stirred at 500 rpm for 8 h until the substances were dissolved. Next, the electrospinning solution was infused into a 10-mL injection syringe. The electrospinning parameters for evenly formed nanofiber membranes included a specified flux rate of 0.25 mL/h, a voltage range of 15–19 KV, a needle movement rate of 30 mm/s, and a rotary speed of 150 rpm for the diverter. After the optimal HPC content was determined, 0.6%, 0.7, and 0.8% *w*/*v* of zinc acetate were separately added to PVP/HPC mixture, and the yielded nanofiber membranes were denoted as PVP/HPC-0.6%, PVP/HPC-0.7%, and PVP/HPC-0.8%. The membranes were compared regarding nanofiber formation and antibacterial efficacy, and the optimal one was applied to wrap the absorbable collagen sutures via the electrospinning technique. Good conditions for electrostatic spraying include the following: core yarn tension must be greater than outer fiber tension and there should be tension and speed difference between core-spun yarn and clad fiber. When the electrostatic spinning machine starts pressuring, the polymer solution passes through the metal needle. This causes the polymer solution to be unstable as a result of introducing an electric charge onto the polymer droplet. At the same time, the charge-repulsive force, contrary to the surface tension of the polymer solution, pulls the solution to form a jet. Finally, the polymer solution flows along the direction of the electric field. The increase in the electric field deforms the spherical droplet into a cone, forming a Taylor cone. The microfibers are deposited from the conical polymer droplets and wrapped onto the core yarn. Internal and external charges cause the droplet jet to wobble in the direction of the collector. Polymer chains in the solution stretch and slide over each other, pressurizing to form nanofibers. The best results of this experiment are produced by changing the positive and negative voltage to 9 KV and controlling the collection speed at 700 rpm, the solution advancing speed at 0.1 mL/h, and the core yarn moving speed at 1 mm/s.

### 2.3. Bacterial Biology Experiment

#### 2.3.1. Bacterial Culture

*Escherichia coli* (*E. coli*, DH5α) and *Staphylococcus aureus* (*S. aureus*, CGMCC1.2465) were used for the bacterial tests. They were all freeze-dried and purchased from the Shanghai Biotechnology Collection Center (SHBCC). To begin, 20 μL of bacterial stock was removed with a pipette, after which a medium (5 mL) was added. Each bacterial strain was cultured in a constant temperature shaker (37 ± 1 °C) at 220 r/min for 24 h.

#### 2.3.2. Antibacterial Test

The evaluation of antibacterial properties mainly refers to the national standard GB/T 20944.3-2008. The experimental strains include Staphylococcus aureus and Escherichia coli. In the early stage of the experiment, bacteria and bacteria solution should be cultured, and fresh bacteria solution should be used as soon as possible to ensure the activity of inoculated bacteria. In the early stage of the antibacterial test, it is necessary to prepare the medium, so bacteria elimination was performed on the culture medium, test tube, and gun head in advance. In the process of preparing the AGAR plate, it is necessary to perform ultra-clean UV elimination on all instruments for more than 15 min to avoid impurity contamination. After the preparation of the AGAR plate, it should be stored in a refrigerator at 4 °C. The liquid in the test tube was diluted through a centrifuge tube, and 100 μL of bacterial liquid was absorbed from the test tube and inoculated on the AGAR plate. Finally, the AGAR plate was spot-coated to make the bacteria grow evenly on the AGAR plate. The AGAR plate was inverted and cultured in a 37 °C oven for more than 18 h before observation.

The culture of a Luria-Bertani (LB) liquid/solid medium was referred to in a previous study [36]. Samples were cultured in a constant temperature shaker at 37 ± 1 °C and 130 r/min for 18 h. Optical density (*OD*) was measured at 600 nm using a multifunctional enzyme marker (Tecan spark 10 M, Switzerland). The yielded *OD* value at 600 nm was recorded and calculated for bacterial concentration according to the following formula. The initial inoculation densities of the two strains ranged from 1 × 10^9^ CFU/mL to 5 × 10^9^ CFU/mL.

*Concentration* (CFU/mL) = *OD*_600_
*×* 10^9^ (CFU/mL)

### 2.4. Cell Biology Experiment

#### 2.4.1. Cell Culture

NIH-3T3 cells (Shanghai iCell Bioscience Co., Ltd., Shanghai, China) were cultured in DMEM and supplemented with a high sugar of 10% calf serum, 1% sodium pyruvate, 1% glutamine, and 1% non-essential amino acids. After the cells grew to 80~90% at 37 °C and 5% CO_2_, they were digested with a trypsin/EDTA solution and re-suspended with high glucose DMEM. The cell concentration was adjusted to 2 × 10^3^ cells and inoculated into 96-well plates for follow-up experiments.

#### 2.4.2. In Vitro Cytotoxicity

The cell activity was quantitatively characterized using the tetrazole salt method. In this method, cells are cultured at a density of 3 × 10^5^ counts/mL and are blown to be evenly distributed. The electrospinning nanofiber membrane is inserted in a 24-well culture plate, followed by 100 μL cell suspension. The culture is conducted in an incubator (37 °C, 5% CO_2_) for four hours; meanwhile, 1 mL of nutrient solution is supplemented. Cells are cultured for one, four, and seven days during which the medium is altered.

The MTT colorimetric analysis is performed as follows to measure the cells’ activity over the samples. When one, four, and seven hours are reached, the 24-well culture plate is removed and 100 μL of MTT solution (5 mg/mL) is added to each well. The cell plate is again cultured in an incubator (37 °C, 5% CO_2_) for four hours. Next, the supernatant is removed and replaced by 300 μL DMSO, after which the culture plate oscillates on a trace amount oscillator for 10 min, which allows the formazan from the cells to be fully dissolved. Finally, 100 μL of the mixture is pulled in from each well, which means that this action needs to repeat three times or once for each well. A multifunctional enzyme marking instrument is used to measure the *OD* with 570 nm of the solution from each well, and the results are compared with TCPS (i.e., the control group).

## 3. Results and Discussion

### 3.1. Morphology Analysis of Nanofibers

**Figure 2:**
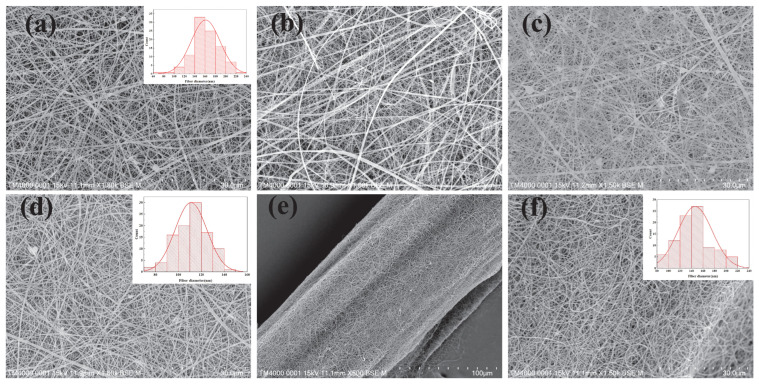
(**a**) SEM images of HPC/PVP electrospun nanofiber membrane; (**b**) SEM images of HPC/PVP-0.06% electrospun nanofiber membrane; (**c**) SEM images of HPC/PVP-0.07% electrospun nanofiber membrane; (**d**) SEM images of HPC/PVP-0.08% electrospun nanofiber membrane; (**e**,**f**) HPC/PVP/Zn electrospun nanofiber membrane, and HPC/PVP/Zn electrospun wrapped yarns.

Figure 2 exhibits SEM images showing the morphology of nanofiber membranes as related to their zinc acetate content. The morphology of HPC/PVP nanofiber membranes exhibit evenly distributed nanofibers with an average diameter of 140–180 nm, and the nanofibers have a sleek surface (Figure 2a). HPC/PVP-0.06% and HPC/PVP-0.07% fiber membranes are not uniformly filamped compared with HPC/PVP-0.08% fiber membranes. The diameter distribution of HPC/PVP-0.08% fiber membranes showed that the filament formation was uniform. Combined with subsequent antibacterial experiments, 0.08%-Zn (*w*/*v*) has the best antibacterial performance, so the optimal concentration was selected as 2%PVP, 6%HPC, 0.08%-Zn (*w*/*v*) (Figure 2b–d). Comparatively, with the incorporation of zinc acetate, HPC/PVP-0.08%Zn nanofiber membranes consist of finer nanofiber than the HPC/PVP group (Figure 2d). The morphology of nanofibers is not correlated with the presence of zinc acetate, and the expected random orientation occurs. Zinc acetate causes gelation formation, which strengthens the viscosity of electrospinning solution, resulting in uneven jet [37]. Afterward, the absorbable surgical sutures are wrapped in the electrospinning solution via the electrospinning process (Figure 2e,f). The SEM image proves that the sutures are evenly covered with nanofibers of a diameter ranging from 120–160 nm. In other words, the evenly distributed nanofibers also substantiate that the electrospinning solution is evenly blended.

### 3.2. FTIR and EDS Analyses

**Figure 3:**
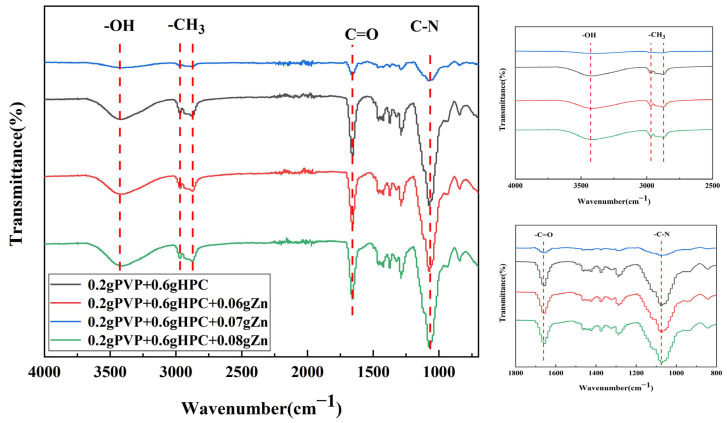
FTIR analyses of nanofiber membranes as related to their concentration of zinc acetate.

The structure of nanofiber membranes containing different zinc acetate contents and the distribution of inorganic Zn ions in the membranes was examined using FTIR and EDS. Figure 3 shows the FTIR spectra of nanofiber membranes related to their zinc acetate content. The characteristic peaks of 3200–3500 cm^−1^ are the -OH extensional vibration of HPC and PVP’s macromolecular chain; characteristic peaks of 2960 cm^−1^ are the -CH_3_ antisymmetric contraction of HPC; characteristic peaks of 2870 cm^−1^ are the contraction of symmetry (-CH_3_) for zinc acetate; and characteristic peaks of 1660 cm^−1^ indicate that C=O where PVP and zinc acetate coexist.

**Figure 4:**
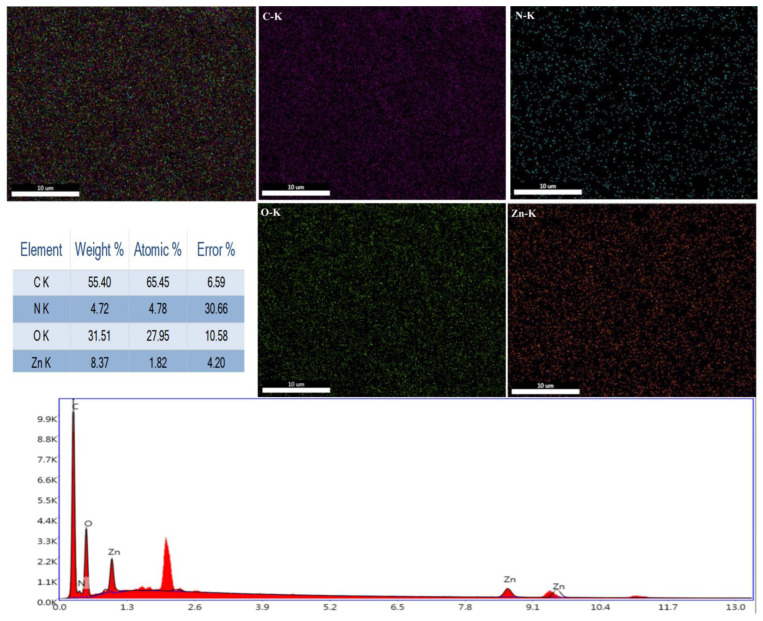
EDS element reflection analyses of PVP/HPC-0.08% nanofiber membranes.

On HPC and PVP, zinc acetate produces a nano-fiber membrane. EDS measurement was conducted to realize the distribution of antibacterial zinc ions in the nanofiber membranes. Figure 4 indicates the element distribution of PVP/HPC-0.08% nanofiber membranes. Energy-dispersive X-ray spectroscopy (EDS) results suggest that zinc elements are better dispersed in nanofiber membranes, and the content is 8.37 wt%. In addition, the correlation peaks of C, N, and O can be observed in the EDS spectrum, and C, N, and O elements can be uniformly distributed on the fibrous membrane. Therefore, zinc elements uniformly distributed on the fibrous membrane coated with the outer layer of surgical sutures can act uniformly and effectively kill bacteria.

### 3.3. In Vitro Antibacterial Activity

**Figure 5:**
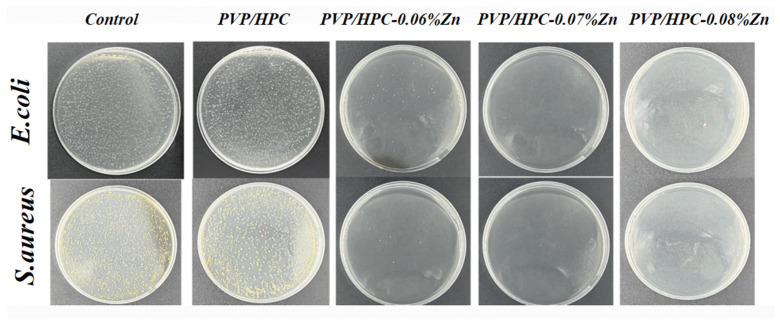
Images of in vitro antibacterial performances against *E. coli* and *S. aureus*.

Clinical surgical sutures lack an antibacterial effect, so an in vitro antibacterial experiment was conducted in order to provide a wound with antibacterial effects. A schematic of the antimicrobial mechanism is shown in Figure 1c. The main antibacterial mechanism of HPC/PVP/Zn nanofiber membranes is the act of Reactive Oxygen Species (ROS) generating oxidative stress on bacteria, leading to cell death [38]. Metal ions are introduced, and metal ions contact microorganisms. Because of the negative charge of the cell membrane and metal ions being subject to gravity, metal ions penetrate the cell membrane, enter the microorganism, and react with the protein in the microbial protein structure destruction. This results in microbial death or dysfunction. Figure 5 displays the zine acetate-contained nanofiber membranes’ quantitative and qualitative antibacterial performance. By contrast, the pure HPC/PVP nanofiber membranes do not exhibit antibacterial efficacy against gram-negative bacterium and gram-positive bacterium, as demonstrated by the evenly growing bacterial strains over the agar plates. With zinc acetate incorporated in HPC and PVP, the resulting nanofiber membranes become highly antibacterial against *E. coli* and *S. aureus*. Compared with fiber membranes containing 0.06% and 0.07% zinc acetate, the one containing 0.08% zinc acetate has a higher antibacterial rate. The antibacterial rate reached 99.9%, and the effect was obvious compared with the control group and the petri dish without zinc acetate. It is substantiated that surgical sutures wrapped in zinc acetate-contained nanofiber membranes can be antibacterial and kill bacteria effectively.

**Figure 6:**
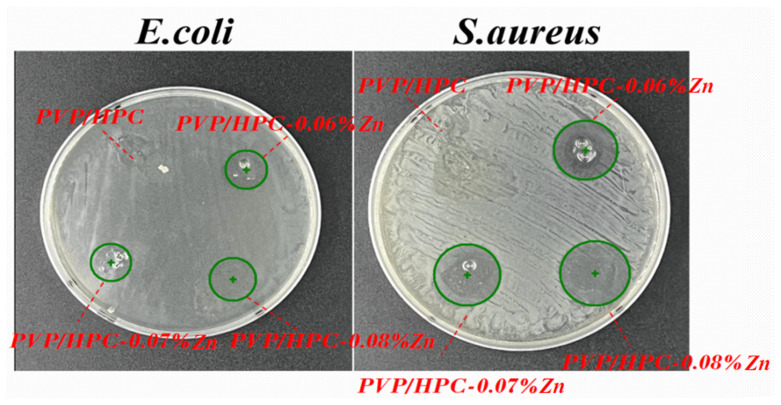
Zone of inhibition for the in vitro antimicrobial activity.

Figure 6 shows the antibacterial efficacy of nanofiber membranes as related to the content of zinc acetate. The HPC/PVP, HPC/PVP-0.06%, HPC/PVP-0.07%, and HPC/PVP-0.08% nanofiber membranes exhibit different levels of antibacterial efficacy based on the zone of inhibition for antimicrobial activity. Samples are trimmed into circular shapes and adhere to the agar spread with *E. coli* and *S. aureus*. The culture is conducted in an incubator with a temperature of 37 °C for 24 h. In particular, the HPC/PVP-0.08% group exhibits a significantly greater diameter of the inhibition zone than other groups, suggesting that a rise in zinc acetate exerts a more significant restriction on the antimicrobial activity.

**Figure 7:**
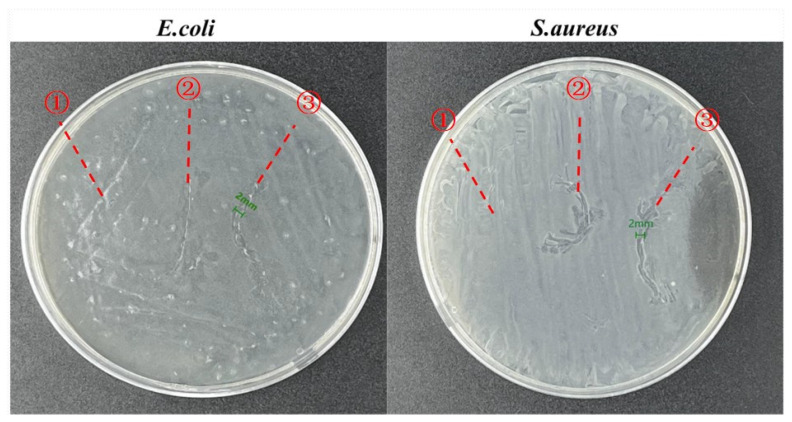
Zone of inhibition for the in vitro antimicrobial activity of ① pure, ② HPC/PVP-coated, and ③ HPC/PVP-0.08% surgical sutures.

To prove that a zinc acetate-containing nanofiber membrane retains antibacterial efficacy when coating surgical sutures, the antibacterial efficacy of surgical sutures wrapped in acetate-containing nanofiber membranes is evaluated in Figure 7. Based on the test results, pure HPC/PVP nanofiber membranes fail to provide the surgical sutures with antibacterial efficacy, specifically surgical sutures wrapped in HPC/PVP-0.08%, which cause a zone of inhibition with a thickness of 2 mm. This result conforms with the antibacterial effect in Figure 6 and indicates that surgical sutures wrapped in HPC/PVP-0.08% demonstrate better antimicrobial activity.

### 3.4. In Vitro Cytotoxicity Analysis

**Figure 8:**
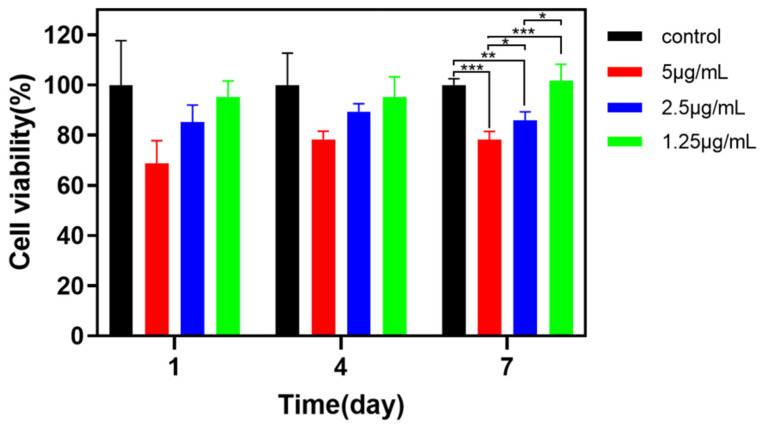
Cell viability of HPC/PVP/Zn nanofiber membranes when co-cultured with NIH3T3 cells. The significance is represented by * (*p* < 0.1), ** (*p* < 0.05) and *** (*p* < 0.01).

Substances coating surgical sutures are required to be non-cytotoxic, which determines the specified group (HPC/PVP-0.08% nanofiber membranes) used for biocompatibility evaluation. As specified in IOS 10993.5, NIH-3T3 cells are used for the in vitro toxicity test, and Figure 8 demonstrates the cell cytotoxicity test results. Compared to the control group, the HPC/PVP-0.08% group adversely affects the cell activity earlier. On day 7, the cell activity is distinctively more significant than it is on day 4, which satisfies the test standard (>70%) [37,39]. The HPC/PVP-0.08% nanofiber membranes show low cytotoxicity and good biocompatibility. Subsequently, this group will be combined with surgical sutures for use in medicine.

**Figure 9:**
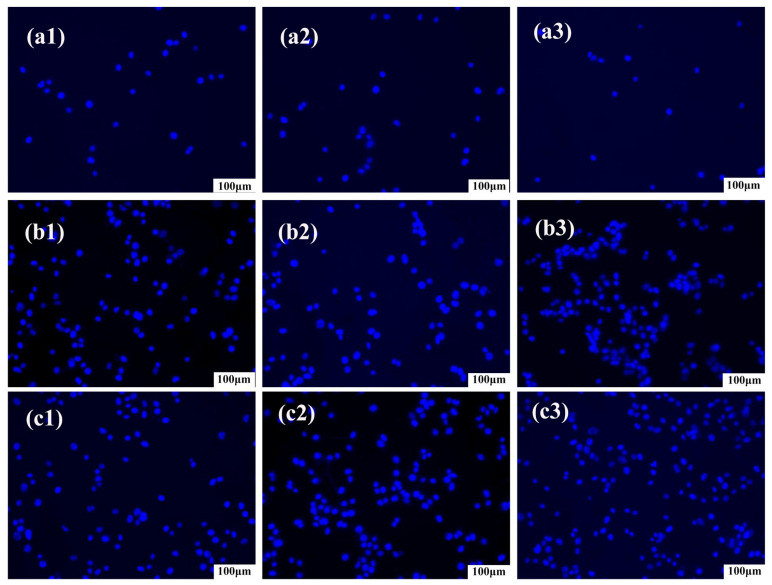
Cell attachment analysis of HPC/PVP/Zn nanofiber membranes ((**a1**–**a3**,**b1**–**b3**,**c1**–**c3**) were cell adhesion maps at 1, 3 and 5 h of cell culture, respectively).

In addition to cell cytotoxicity, cell adhesion is also expected to be achieved in this study after the incorporation of zinc acetate. Therefore, cell attachment is observed in 1, 3, and 5 h as shown in Figure 9. The cells do not grow nor adhere well in the initial first hour; however, with time, cell adhesion is increasingly improved, which suggests that cell growth benefits greatly from a supportive environment with zinc acetate-contained- nanofiber membranes. The experiment proved that the zinc-added fiber membrane has good biocompatibility and can be used in the medical field for wound sutures.

## 4. Conclusions

In this study, a method to produce electrostatically coated yarn was employed to cover absorbable collagen surgical sutures in a nanofiber membrane. SEM observation indicates that HPC/PVP nanofiber membranes are composed of optimal spinning nanofibers when the blending ratio is 6% *w*/*v*:2% *w*/*v*. Furthermore, zinc acetate was combined with HPC and PVP to form nanofiber membranes that were then proven to have comparatively more even spinning nanofibers. There was no apparent beading on the surface. Additionally, SEM images show that absorbable collagen surgical sutures can be easily wrapped in a nanofiber membrane. Next, FTIR and XRD were performed to examine whether zinc acetate is loaded over the nanofiber membranes, which was then proven correct. Moreover, an antibacterial assay was performed to examine whether the nanofiber membranes are antibacterial compared with the control group and the fiber film without zinc acetate, and the results indicate that nanofiber membranes containing zinc acetate demonstrate excellent antibacterial efficacy at 99.9% regardless of whether the bacterium is *E. coli* or *S. aureus*. In addition, an antibacterial zone test was carried out on the fiber membrane with zinc acetate added. An antibacterial zone test was also carried out on the surgical sutures wrapped with zinc nanofiber membrane. The experimental results show that the fiber membrane prepared using electrostatic wrapping technology had good antibacterial effects after being covered in the surgical sutures. Finally, an MTT assay and cell adhesion evaluation were employed to determine that zinc acetate-contained nanofiber membranes exhibit low cytotoxicity and good biocompatibility. Compared to the control groups, the zinc acetate-contained nanofiber membranes demonstrate increasingly improved cell adhesion, which proves that the nanofiber membranes containing zinc acetate provide a suitable and supportive environment for cell growth with time. In conclusion, the zinc-loaded suture prepared using the electrostatic coating technique has an antibacterial effect and can be applied in the biomedical field.

## Figures and Tables

**Table 1:** Formulation of HPC/PVP/Zn nanofiber membranes.

Number of Samples	Strength of Solution			Voltage (KV)
	PVP (*w*/*v*)	HPC (*w*/*v*)	Zn (*w*/*v*)	
1	1%	5%	-	19
2	1%	6%	-	19
3	1%	7%	-	19
4	2%	6%	0.6%	19
			0.7%	
			0.8%	
5	2%	7%	-	19
6	2%	8%	-	19
7	2%	10%	-	19

## Data Availability

1. The data presented in this study are openly available in [repository name e.g., FigShare] at [doi], reference number [reference number]. 2. The datasets used and/or analysed during the current study available from the corresponding author on reasonable request. 3. The data are not publicly available due to restrictions apply to the availability of these data.Data are however available from the authors upon reasonable request and with permission of [third party]. 4. No new data were created or analyzed in this study. Data sharing is not applicable to this article. 5. All data generated or analysed during this study are included in this published artiale.
